# Baked Egg Oral Immunotherapy: Current State in Pediatric Age

**DOI:** 10.3390/nu16183203

**Published:** 2024-09-22

**Authors:** Simone Foti Randazzese, Lucia Caminiti, Mariarosaria La Rocca, Cristina Italia, Fabio Toscano, Francesca Galletta, Giuseppe Crisafulli, Sara Manti

**Affiliations:** Pediatric Unit, Department of Human Pathology in Adult and Developmental Age “Gaetano Barresi”, University of Messina, Via Consolare Valeria, 1, 98124, Messina, Italylcaminiti@unime.it (L.C.); crisafulli.giuseppe@unime.it (G.C.)

**Keywords:** baked egg, children, hen’s egg allergy, oral immunotherapy, review

## Abstract

Hen’s egg allergy is one of the most common food allergies in the Western world, with an increase in recent years. It affects about 9.5% of the pediatric population, and the onset most often occurs before the first year of life. The occurrence of spontaneous oral tolerance acquisition varies among studies, but it is generally high by school age. Nowadays, allergen immunotherapy may represent the only therapeutic strategy able to modify the natural history of hen’s egg allergy. Specifically, many children with hen’s egg allergy may tolerate baked eggs. Food processing, specifically high temperatures, alters the allergenicity of hen’s egg proteins by causing conformational changes in allergen epitopes, which makes them less allergenic. This review aims to discuss the scientific evidence in the field of baked egg oral immunotherapy in hen’s egg-allergic children, with a meticulous examination of the pertinent literature surrounding the subject matter.

## 1. Introduction

Nowadays, food allergy (FA) represents a significant public health problem, especially in the pediatric population, with an increase in prevalence and severity [1]. Ingesting food antigens may cause an adverse reaction to food mediated by different immunologic mechanisms, including immunoglobulin E (IgE), cell-mediated, and both IgE and cell-mediated mechanisms [2]. Clinical manifestations typically occur after the ingestion of the allergen and may vary from mild to severe, including skin (e.g., urticaria, eczema, itching, and/or angioedema), respiratory (e.g., rhinorrhea, sneezing, cough, and/or wheezing), or gastrointestinal (e.g., abdominal pain, nausea, vomiting, and/or diarrhea) reactions. In severe cases, these reactions may progress to anaphylaxis, a life-threatening allergic reaction that requires emergency medical treatment [3].

More than 160 foods may cause FA, with varying prevalence rates by the specific food and population affected [4]. Specifically, hen’s egg (HE) is a ubiquitous food, consumed in most parts of the world. It is cheap and easily accessible, used in many homemade dishes, and it is also widely used by the food industry in processed foods. HE allergy (HEA) is one of the most common IgE-mediated FAs in pediatric ages, with a typical onset in the first year of life [5].

Managing HEA and, in general, FA involves an avoidance diet, emergency treatment, and education of family and caregivers [6]. This impacts quality of life (QoL) negatively and creates high costs for the healthcare system, resulting in significant morbidity and psychosocial burden [7,8]. Therefore, looking for prevention strategies and/or resolutive therapies is imperative.

Currently, the only active strategy able to modify the natural history of allergies is represented by allergen immunotherapy (AIT) [9,10,11,12,13]. In the field of FA treatment, there are several different forms based on the route of administration, including oral (OIT), sublingual (SLIT), subcutaneous (SCIT), and epicutaneous immunotherapy (EPIT) [14]. Specifically, OIT showed the most promising results in terms of efficacy, but with significant rates of adverse reactions, primarily gastrointestinal [15]. It is well-established that many children with HEA may tolerate extensively baked egg (BE), such as cakes or muffins [16]. The high baking temperatures alter HE proteins’ structure, reducing their allergenic potential [17]. To date, OIT with BE represents a treatment approach for children with HEA that involves gradually introducing BE products into their diet, with the aim to increase the tolerance to HE proteins and potentially reduce the severity of their allergic reactions [18].

This review aims to discuss the current state of OIT with BE in the pediatric population with HEA across a meticulous analysis of key papers. We performed a literature search in Medline through PubMed using keywords such as “baked egg” and “oral immunotherapy” and “children” or “pediatrics”. Original studies and review articles, with a focus on meta-analyses and randomized controlled trials (RCTs) in English, were identified up to January 2023.

## 2. Epidemiology

HEA is one of the most common FAs in the Western world, with an increasing rate of prevalence in the last decades [19]. It affects about 9.5% of children, with a usual onset before the first birthday and a 1.2–2.9% prevalence at 2 years of age [20,21].

Motosue et al. conducted an observational study using a national administrative claims database from 2005 to 2014. During the study period, participants under 18 years old experienced 7310 food-induced, anaphylaxis-related emergency department visits. Cow’s milk (CM) and HE were more frequent triggers among very young children (0–2 years) compared to older preschool and school-aged children (≥3 years) [22].

These data were confirmed by Grabenhenrich et al., whose analysis of the European Anaphylaxis Registry showed CM and HE as the most common triggers in children under six years old [23].

Recently, Spolidoro et al. provided 10-year updated prevalence estimates for the so-called “big eight” FAs. The review included 93 studies published between 2012 and 2021, with 67 studies focusing solely on children. The overall estimates for all age groups of self-reported lifetime prevalence were as follows: CM (5.7%), HE (2.4%), wheat (1.6%), soy (0.5%), peanut (1.5%), tree nuts (0.9%), fish (1.4%), and shellfish (0.4%) [24].

The prevalence and age of tolerance acquisition among children vary due to population differences and the means of diagnosis [25]. It is estimated that 50% of children develop tolerance in the first 2–3 years of life and up to 80% by school age [26]. Tolerance to BE is achieved more quickly, with 94% of patients outgrowing BE allergy by age 12 [27]. However, especially children with higher egg white (EW) and Gal d 1 specific IgE (sIgE) and/or a history of anaphylaxis may develop a persistent HEA [28].

## 3. Prevention of Hen’s Egg Allergy

HE introduction into the diet may represent a critical moment for children and their families. HEs are present in a wide variety of foods, including pasta, cakes, ice cream, and biscuits, making them difficult to avoid and increasing the risk of accidental exposure. Several allergic reactions occur during the first known exposure to HE [29].

HEA risk factors may be genetic and environmental, such as reduced microbial exposure, reduced serum vitamin D levels, and compromised skin barrier in children with atopic dermatitis (AD) [30,31,32].

AD is frequently related to FA, especially to HEA, CM allergy (CMA), and peanut allergy (PA), possibly due to skin barrier disruption [32]. In the EuroPrevall birth cohort, 12,049 infants were recruited, and 9336 (77.5%) were followed until age two. Eczema was strongly associated with the development of HEA, with the association becoming more robust as the severity of eczema increased [32].

There is also a higher risk of HEA developing in children with a family history of atopy [33].

Gut microbiota may play a role in HEA. Fazlollahi et al. enrolled 141 children with HEA and controls from the Consortium of Food Allergy Research study. Fecal samples were collected from children aged 3 to 16 months. Clinical evaluations, skin prick tests (SPTs), and sIgE were performed. The gut microbiome was analyzed using 16S rRNA sequencing. Analyses focused on HEA and HE sensitization at enrollment and the resolution of HEA by 8 years of age. Compared to controls, children with HEA had distinct gut microbiota. Specific genera from *Lachnospiraceae*, *Streptococcaceae*, and *Leuconostocaceae* families were more abundant in HE-allergic children. Functional analyses indicated differential purine metabolism in the gut microbiota of this cohort of subjects. Greater gut microbiome diversity and genera from *Lachnospiraceae* and *Ruminococcaceae* were linked to HE sensitization. Identifying distinct early-life gut microbiota in HE-allergic and HE-sensitized children, the study suggests potential targets for preventive or therapeutic interventions in children with HEA [34].

More recently, Yamagishi et al. documented that children with HEA may experience dysbiosis characterized by decreased levels of butyric acid, produced by butyric acid-producing bacteria in the intestine, which promotes the maturation of regulatory T cells (Tregs) [35].

Instead, there is no evidence to suggest that avoiding HE during pregnancy or breastfeeding reduces the risk of developing HEA [36].

The timing of introduction into children’s diets represents a vital role in developing HEA. According to the leading European and American scientific organizations of allergy and immunology recommendations, HE should be introduced into the diet at an early age, between 4 and 6 months of life [37,38]. Indeed, the elimination of HE may expose the child to severe nutritional deficiencies and failure to thrive, especially in the early stages of life, associated with a negative psychosocial impact [37]. Furthermore, children who have not consumed HE and/or food containing it for an extended period may develop reactions [39].

## 4. The Role of Component-Resolved Diagnosis in Hen’s Egg Allergy

A proper diagnosis of HEA is essential for improving effective preventive strategies to avoid potentially fatal conditions, such as anaphylaxis, and for assessing subjects who may be re-exposed to the foods [40].

The diagnosis is based on the integrated analysis of the clinical history and the results of diagnostic tests [41]. The onset of signs and symptoms immediately after the ingestion of baked, cooked, or raw HE or food containing HE is highly suggestive of an IgE-mediated allergic reaction [41].

Among diagnostic tests, SPTs with commercial extracts, prick by prick (PbP) with raw egg (RE), and/or the determination of sIgE are commonly employed in clinical practice [42]. Both tests have good sensitivity but low specificity. As a result, an Oral Food Challenge (OFC) remains the gold standard for diagnosing HEA, despite the challenges posed by the potential risk of patients developing a severe anaphylactic reaction during the test [43].

It is well known that larger SPT wheal sizes and higher sIgE increase the likelihood of allergic clinical manifestations [44]. Calvani et al. systematically reviewed the literature to determine recommended cutoffs for SPTs and sIgE for both EW and major HE allergens. HEA is highly probable in children under 2 years when SPTs with EW extract are ≥4 mm or sIgE are ≥1.7 kUA/L. In children aged 2 years and older, an OFC may be unnecessary if SPTs with EW extract are ≥10 mm or PbP with EW is ≥14 mm, or if sIgE are ≥7.3 kUA/L. Similarly, heated HEA is highly probable if SPTs with EW extract are >5 mm for children under 2 years and >11 mm for those aged 2 years and older [44].

Nevertheless, all the guidelines emphasize that SPTs and/or sIgE positivity are not contraindications of OFC, and their negativity does not exclude a positive OFC [45].

Moreover, the emergent role of Component-Resolved Diagnosis (CRD) is currently critical [46]. CRD may improve allergy management, which is particularly evident for FA. Identifying the specific allergenic molecules to which a patient is sensitized can help determine the likelihood of local vs. systemic adverse reactions during an OFC. It also clarifies the actual sensitization profile of multi-sensitized patients, predicts the persistence of clinical manifestations, and establishes the stability of the allergen to heat and digestion [47]. Allergens that are stable to heat and digestion are more likely to cause severe clinical reactions. In contrast, heat- and digestion-labile molecules are more likely to cause mild and/or local reactions or to be tolerated [48]. Moreover, determining whether sensitization is genuine or due to cross-sensitization may help to evaluate the likelihood of reaction upon exposure to different allergen sources [47].

Specifically, HE is composed of water (76%), proteins (12%), and lipids (9.51%), with the yolk comprising 27.5%, EW 63%, and eggshell and membranes 9.5% [49]. One HE contains approximately 6.6 gr of proteins [49]. Five proteins most commonly involved in allergic reactions to HE were identified and characterized [50]. The main characteristics of Gal d 1, Gal d 2, Gal d 3, Gal d 4, and Gal d 5 are reported in Table 1 [50].

Gal d 1 (ovomucoid) is present in a lower quantity (11%) in EW than Gal d 2 (ovalbumin) (54%), but it is considered the immunodominant HE allergen [51].

In addition, Gal d 2 and Gal d 3 (ovotransferrin) are heat- and digestion-labile, while Gal d 1 is characterized by heat- and digestion-stability and may cause adverse reactions after ingestion of all forms of HE [46]. Specifically, the denaturation temperature of Gal d 2 and Gal d 3 is estimated to be approximately 60–70 °C, while Gal d 1 requires higher temperatures (approximately 90 °C) to be denatured [52]. This is a crucial aspect, as subjects who have CRD positivity for Gal d 2 and Gal d 3 are less likely to experience adverse reactions after consuming BE, in contrast to those who are positive for Gal d 1, who may more likely exhibit clinical manifestations even after the ingestion of BE [51].

Furthermore, the allergenicity of BE may also be reduced by the allergen epitopes’ interaction with the food matrix, such as wheat flour, which may block the epitopes’ access [53].

Recently, Liu et al. aimed to define how food matrices impact the matrix effect in HE allergenicity. The enzyme-linked immunosorbent assay (ELISA) was used to quantify Gal d 1 and Gal d 2 in extracts from various products containing BE. Allergen-specific IgE-blocking ELISA tests were conducted using HE product extracts on patients with HEA to determine if each extract’s amount of HE protein is associated with the extracts’ ability to bind to patients’ HE-sIgE. When HEs were baked in any muffin matrix, there was an observed increase in the amount of extractable Gal d 2, while the amount of extractable Gal d 1 decreased compared to plain BE. Among the different muffin types, rice-based muffins yielded a higher amount of extractable Gal d 2 than wheat muffins, whereas muffins made with a combination of wheat and banana had elevated levels of both Gal d 1 and Gal d 2. The HE allergens in the extracts were able to block HE-allergic patients’ HE sIgE, indicating a potential impact on allergenicity. In conclusion, structural modification of HE allergens might allow safe consumption of BE-containing foods [54].

Currently, understanding molecular sensitization profiles may help identify patients who are eligible for AIT [55].

## 5. Principles of AIT: Desensitization vs. Tolerance

AIT involves repeated exposure to allergens at different intervals. The goal is to modulate the immune response, thereby reducing signs and symptoms and the need for medication for allergic reactions, as well as preventing the development of new allergies [9].

Continuous exposure to allergens during AIT induces several changes in the immune response [56]. AIT promotes a shift from a T helper 2 (Th2) to a Tregs and a T helper 1 (Th1) response [57]. Specifically, Th1 cells produce interferon-γ (IFN-γ), which counteracts the effects of Th2 cytokines, such as interleukin-4 (IL-4), interleukin-5 (IL-5), and interleukin-13 (IL-13), that are involved, e.g., in the production of IgE and the activation of eosinophils. Moreover, Tregs produce immunosuppressive cytokines, such as interleukin-10 (IL-10) and Transforming Growth Factor-β (TGF-β), which inhibit the activity of Th2 cells, contributing to the immune regulation [58]. The increased Tregs activity also influences B cell responses, increasing allergen-specific IgG4, which block the interaction between allergens and IgE. This reduces IgE levels over time and prevents mast cell and basophil activation, making these cells less responsive to allergen stimulation. Consequently, there is a decreased release of histamine and other inflammatory mediators [59]. Furthermore, AIT causes a downregulation of the high-affinity IgE receptor (FcεRI) on mast cells and basophils, which reduces their potential activation [60].

The primary goal of AIT in patients with FA is to induce desensitization, which refers to the improvement in OFC outcomes by relying on continued exposure to the allergen. On the other hand, tolerance or sustained unresponsiveness is a long-term state of non-reactivity to the allergen that persists even without exposure [61]. Although desensitization may be achieved in most cases, most patients lose the effect after interruption of food intake [61].

AIT is characterized by a build-up phase, which generally takes place in the hospital, where the dose of allergen administered is gradually increased, followed by a maintenance phase [1].

The maximum tolerated dose is administered at home until the increase of the next dose, for example, once a week or every 2 weeks. However, there are several differences in protocols regarding, e.g., duration, dosage, and ways of administration [1].

AITs for FA currently under study include OIT, SLIT, EPIT, and SCIT [14]. In the last decade, new modalities that may enhance the efficacy and safety of AIT were explored, including the use of probiotics (i.e., *Lactobacillus*), monoclonal antibodies (i.e., coadministration of omalizumab or dupilumab), and nucleic acid vaccines [1,61]. These may modulate the immune response and help manage signs and symptoms when administered with OIT [1,61].

Finally, there are absolute and relative contraindications to AIT [9]. Absolute contraindications include poor adherence, uncontrolled asthma, active autoimmune diseases, neoplastic diseases, or eosinophilic esophagitis (EoE) [9]. Among the relative contraindications, there are severe AD, chronic spontaneous urticaria, cardiovascular diseases, and treatment with β-blockers or Angiotensin-Converting Enzyme inhibitors [9].

## 6. Baked Egg Oral Immunotherapy

According to the guidelines of the European Academy of Allergy and Clinical Immunology (EAACI), OIT may be recommended in children with persistent HEA from around 4–5 years of age [9]. OIT is known to be associated with considerable rates of adverse reactions, including, more frequently, local reactions such as itching of the oropharynx and perioral rash, as well as, less frequently, systemic reactions like abdominal pain, vomiting, wheezing, urticaria, and anaphylaxis [62,63]. 

Several real-world data demonstrated that performing OIT early, especially during preschool age (<6 years old), is significantly safer and more effective compared to starting in older children [64]. Indeed, infants develop fewer allergic reactions involving the respiratory, cardiovascular, and neurological systems compared to older children [65]. 

Improving the tolerability of OIT is an essential goal [66]. The most common approach is represented by the reduction of the allergenic power through thermal treatments [67]. Indeed, BE may reduce the adverse reactions of OIT and may be better tolerated, with regular ingestion helping accelerate the resolution of HEA, improving the QoL of the child and their family [68]. Several studies, summarized in chronological order in Table 2, were conducted in the last decade [69,70,71,72,73,74,75,76,77].

In 2016, Bravin and Luyt conducted a clinical trial with the development of a recipe containing HE to be used in BE OIT, aiming to evaluate its safety and efficacy in a home-based, gradual up-dosing regimen for 15 children with persistent IgE-mediated HEA (SPT to EW and EY > 3 mm) and an allergic reaction to BE in the previous 6 months or a positive open OFC result. Starting with an initial dose of 125 μg of HE protein, the dosage was increased daily over 60 days, reaching a maximum target dose of 6.25 g of HE protein. Once the maximum dose was achieved, the patients underwent an OFC with boiled HE to determine whole HE tolerance. In total, 8/15 (53%) patients successfully completed the OIT program: four (50%) within the 60-day protocol target without adverse reactions and four (50%) between 80 and 270 days. Of the seven remaining patients, two could not tolerate the initial dose and continued strict HE-free diets, while five achieved partial tolerance between days 10 and 47, allowing them to incorporate trace amounts of HE into their diets. All adverse reactions were mild and managed with oral antihistamines when necessary [69]. 

Gotesdyner et al. conducted a case-control study to evaluate the effectiveness and safety of a structured graduated exposure protocol (SGEP) using extensively heated baked egg (EHBE) to promote tolerance in 39 children under 2 years old with IgE-mediated HEA diagnosed by OFC or by positive SPT and/or positive IgE along with a clinical history of an immediate allergic reaction after exposure to cooked (CE) or fried egg (FE) in the past year. These subjects were compared to a control group of 80 children with IgE-mediated HEA diagnosed by OFC or by positive SPT and/or positive IgE along with a clinical history of an immediate allergic reaction after exposure to HE, who followed a strict avoidance diet. The median age for HEA resolution in the SGEP group was 24 months, compared to 78 months in the control group (*p* < 0.001). By the final follow-up, 82% of children in the SGEP group were tolerant to lightly cooked egg (LCE), compared to 54% in the control group (*p* = 0.001). Anaphylactic reactions were reported in 50% of the children in the intervention group and 32% of the children in the control group (*p* = 0.068). More than 70% of the subjects in both groups developed skin manifestation as part of their initial reaction (*p* = 0.481) [70]. 

Bird et al. conducted a study to determine if children allergic to BE could become desensitized to LCE after approximately two years of BE OIT. The study included 13 children aged 1 to 18 years with HEA who had either reacted during a BE OFC with 3.8 g of HE protein or had Gal d 1 sIgE > 50 kUA/L. Participants followed a daily BE ingestion protocol, starting with small amounts and gradually increasing to 3.8 g. Eight subjects completed one year of BE OIT, and 7 subjects passed the 3.8 g BE OFC. After an additional year of daily 3.8 g BE ingestion, 5 out of 6 children successfully passed an OFC with 6 g of LCE. The study observed significant decreases (*p* < 0.05) in the ratios of IgE to IgG4 for EW, Gal d 1, and Gal d 2 allergens by 6 months, and these ratios remained low. Levels of IgG4 for these allergens significantly increased by 6 months and stayed elevated. Gal d 2 sIgE significantly decreased (*p* < 0.05) at 12 and 24 months, while the EW SPT wheal diameter significantly decreased (*p* < 0.05) at 12 and 18 months. However, there were no significant changes in sIgE for EW and Gal d 1. The treatment protocol was well-tolerated, with only 1.37% mild adverse reactions reported and none requiring epinephrine [71].

Pérez-Quintero et al. conducted a retrospective analysis to determine if daily ingestion of BE accelerates tolerance to RE compared to those who either avoid BE or initially do not tolerate it. Seventy children diagnosed with IgE-mediated HEA from 2008 to 2014, followed up until 2018, were enrolled. The diagnosis was based on clinical history, SPT, and/or detectable (>0.35 kUA/L) sIgE to EW, Gal d 1, or/and Gal d 2. Since 2010, BE-tolerant children have been encouraged to consume BE daily. Tolerance to BE was established through clinical history or an OFC. Annual OFCs were conducted to assess tolerance to cooked and RE. At diagnosis, 33/70 (47%) patients tolerated BE and continued its daily ingestion, 16/70 (23%) tolerated BE but avoided it, and 21/70 (30%) did not tolerate BE. Patients tolerating BE at diagnosis and consuming it daily achieved tolerance to RE after 27 months, significantly earlier (*p* < 0.05) than those who avoided BE (58 months) or those who did not tolerate BE initially (54 months). Patients avoiding BE had similar outcomes to those initially not tolerating it. Moreover, BE-tolerant patients showed significantly lower sIgE to Gal d 1 and Gal d 2 compared to BE non-tolerant patients (*p* < 0.05) [72].

Machinena et al. aimed to evaluate tolerance to BE in HE-allergic children younger than six years and to assess if regular BE consumption aids in developing tolerance to LCE and RE. A prospective study was conducted on children aged 12 months to 6 years with suspected HEA, diagnosed based on clinical history, SPT > 3 mm, and/or sIgE > 0.35 kUA/L. Children underwent OFCs with extensively heated egg (EHE) and BE. Participants were divided into BE-tolerant and non-BE-tolerant groups based on their OFC results. Of 60 children, 56/60 (93.3%) completed the BE-OFC: 36/56 (64.3%) were BE-tolerant; 20/56 (35.7%) were non-tolerant. The BE-tolerant group was advised to consume up to 1.1 g of BE protein daily, while the non-tolerant group continued a strict HE avoidance diet. The study showed that EW-sIgE ≥ 3.38 kUA/L and Gal d 1 sIgE ≥ 3.51 kUA/L are potential markers to predict BE reactivity with 80% sensitivity and 63.9% and 72.2% specificity, respectively. BE-tolerant children showed significantly lower sIgE than non-tolerant children in terms of HE components (*p* < 0.05). In the non-tolerant group during BE-OFC, 70% developed urticaria, 60% experienced gastrointestinal clinical manifestations, 20% had rhinoconjunctivitis, and 45% had anaphylaxis, with one case involving the lower respiratory tract. The study concluded that 64.3% of HE-allergic children under six could tolerate BE. A supervised OFC remains essential due to the risk of severe reactions [73].

Gruzelle et al. evaluated the efficacy and safety of BE OIT in 71 children with HEA and high sIgE to EW and Gal d 1 following a low-dose BE OIT. These children underwent a total of 164 OFCs, comprising 71 BE challenges and 93 unbaked HE challenges. Most children were treated with BE OIT. A total of 56/71 (78.9%) subjects in the study did not react to the first low-dose BE OFC (702 mg of protein). However, 8.7% of the children discontinued OIT at home. Ultimately, 47/71 (66.2%) children achieved desensitization to unbaked HE vs. 24/71 (33.8%) subjects who did not. Additionally, children with lower EW-sIgE had a significantly higher probability of becoming desensitized to HE (baseline mean EW sIgE 18 vs. 30 kUA/L, *p* = 0.048). Regarding the safety profile, 10/71 (14.1%) children had reactions during OIT (primarily, gastrointestinal events), with 6/71 (8.7%) discontinuing OIT at home; epinephrine was never used. In conclusion, most children with HEA and high sIgE to EW and Gal d 1 tolerated the introduction of BE [74].

Thomas et al. aimed to evaluate the safety, user satisfaction, rates of tolerance achieved at home, and potential barriers of using a structured HE ladder for introducing HE into the diets of 47 children and adolescents (aged 0–18 years old) with mild-to-moderate HEA and no history of anaphylaxis. An HE tolerance ladder, designed for the gradual home introduction of BE and LCE, was used. Most patients (41/47, 87.2%) started the HE ladder at home, while only 3/47 (6.4%) used inpatient resources. Patients spent an average of 15.5 months on the ladder. At the time of review, 20/47 (43%) had completed the HE ladder, though some parents still believed their child was allergic to HE. A total of 18/47 (38.3%) patients reported mild skin reactions, with one severe reaction requiring adrenaline. SPT size was a poor predictor of reactions. High satisfaction was reported, with 78.7% of parents satisfied or very satisfied with the HE ladder. The main barrier was the taste and texture of HE products. In conclusion, the structured HE ladder demonstrated as a safe, well-tolerated, and positive approach for introducing HE into the diets of children with mild-to-moderate HEA. It reduced the need for inpatient challenges, saving resources in busy allergy clinics [75].

De Vlieger et al. aimed to determine the optimal duration for gradually inducing HE tolerance at home in children who may tolerate BE, with the goal of achieving complete RE tolerance. Children over 12 months of age with IgE-mediated HEA, defined by a history of a type I hypersensitivity reaction to HE and a positive HE SPT (wheal > 3 mm) and/or detectable HE sIgE (≥0.35 kUA/L), who tolerated BE, were randomly assigned to a short- or long-duration protocol for HE tolerance induction. The short protocol lasted 18 months, while the long protocol extended to 30 months. The stepwise introduction started with BE (cake), followed by hard-boiled HE, omelet/waffle/pancake, soft-boiled HE, and finally RE. Only children who successfully passed an in-hospital cake challenge or had Gal d 1-sIgE ≤ 1.2 kUA/L, considered safe for introduction at home, were included. Gel electrophoresis showed that heating weakened the Gal d 2 band but left the Gal d 1 band stable. IgE binding to Gal d 2 decreased with heating, whereas Gal d 1’s IgE reactivity was reduced when heated with wheat. Of the 78 children in the study, 39/78 (50%) were assigned to each protocol arm. A total of 58/78 (74.3%) children achieved RE tolerance, with 80% in the short-duration group and 69% in the long-duration group. The median time to reach RE tolerance was 24 months in the short-duration group and 30 months in the long-duration group (*p* = 0.005). No grade IV reactions or cases of EoE were observed. The study concluded that a gradual, short-duration protocol is safe and allows clinicians to guide BE-tolerant children towards RE tolerance at home. Furthermore, it confirmed that HE protein allergenicity is influenced by high temperature, heating duration, and the presence of wheat [76].

Kotwal et al. conducted a retrospective review to identify factors that may predict future HE consumption and barriers to progression based on characteristics observed during and after BE OFC. The study involved 243 patients with a minimum follow-up of 24 months, with target doses ranging from 1/16 to 1/4 of an HE. Outcomes were categorized as pass (no reaction), fail (with BE introduction allowed), or fail (avoidance recommended). Of the participants, 134/243 (55%) passed the BE OFC, while 109/243 (45%) failed, with 70/109 (64.2%) of the failed group advised incorporating BE into their diet. At follow-up (median 47 months), 90/243 (37%) consumed regular HE, 26/243 (11%) ate LCE, 39/243 (16%) consumed BE, and 88/243 (36%) avoided HE entirely. Among those who failed, 58% were consuming some form of HE, compared to 81% of those who passed. The review also found that median HE IgE levels were significantly higher among avoiders compared to those who introduced HE into their diet (median 8.7 vs. 5.8 kUA/L, *p* = 0.008). Lower HE IgE levels and younger age were predictors of some form of HE consumption at follow-up (median IgE 5.8 vs. 8.4 kUA/L, *p* = 0.030; median age 4.0 vs. 8.0 years, *p* < 0.001). A total of 136 reactions were reported among 94/204 (46.1%) patients instructed to introduce some amount of BE at home, with 132/136 (97.1%) classified as mild and 4/136 (2.9%) as severe. These reactions occurred during accidental exposures (22/136, 16.2%), planned dose escalations (42/136, 30.9%), and doses that had been previously tolerated (72/136, 52.9%). Gastrointestinal reactions were the most common (78/136, 57.4%), followed by oral symptoms (48/136, 35.3%), skin reactions (27/136, 19.9%), and upper respiratory symptoms (5/136, 3.7%). Most patients who underwent a BE OFC continued to consume some form of HE, often progressing to direct HE consumption [77].

## 7. Certainty and Uncertainty in Baked Egg Oral Immunotherapy

Balancing certainty and uncertainty in BE OIT requires careful consideration of each child’s individual case, risk factors, and close monitoring throughout treatment. Although the therapy offers a promising path toward tolerance, the variability in outcomes and the potential for allergic reactions must be accounted for in decision-making.

Regarding certainty, the studies mentioned above showed that BE OIT can help some children develop tolerance to HE proteins. Gradual exposure to EHBE appears to induce desensitization, allowing many children to safely consume BE and potentially other forms of HE over time. Furthermore, current research indicates that starting with BE, where the proteins are denatured and less allergenic, is generally safer and more tolerated than RE or LCE. This approach lowers the risk of severe reactions compared to sudden exposure to uncooked HE. Finally, successful BE OIT can reduce anxiety for families and offer children a more normal diet. Some children even go on to tolerate other forms of HE, such as scrambled or boiled.

Regarding uncertainty, it is well-known that not all children respond to BE OIT in the same way. While some can develop tolerance, others can still react to even small amounts of BE or can take longer to see results. Heterogeneity in protocols may also represent a limit: different studies use varying BE OIT protocols, including differences in the starting doses, duration of treatment, and methods for assessing tolerance. This makes it difficult to compare outcomes across studies and establish standardized recommendations for clinical practice, as well as the lack of reliable biomarkers for predicting success. Finally, the long-term outcomes are still under study. It is uncertain whether tolerance to BE will lead to tolerance to all forms of HE or if some children may relapse into allergy after stopping treatment.

## 8. Conclusions

BE OIT represents an effective and relatively safe method to induce desensitization in children with HEA. It involves the gradual introduction of BE products, which are less allergenic than RE or LCE. This approach was shown to increase the likelihood of developing tolerance to both baked and regular HE over time. While the risk of adverse reactions exists, it is generally lower compared to traditional OIT with HE, making BE OIT a promising therapeutic option. Proper medical supervision, emergency preparedness, and regular monitoring are essential to ensure safety and maximize the efficacy of this treatment. However, international research based on high-quality clinical trials with long-term evaluations represents a priority in enriching the amount of evidence currently available to implement this treatment in clinical practice.

## Figures and Tables

**Table 1:** Classification of the major hen’s egg allergens modified from *EAACI Molecular Allergology User’s Guide 2.0*, 2023 [50].

Protein Name	Molecular Weight (kDa)	Protein Family	Biological Functions	Heat Stability	Clinical Relevance
Gal d 1 (Ovomucoid)	28	Kazal-type serine protease inhibitor	Serine protease inhibition and antibacterial activity	High	Heat-stable and highly allergenic. Risk for reaction to all forms of HE.
Gal d 2 (Ovalbumin)	45	Serine protease inhibitor	Storage protein?	Low	Heat-labile. Most abundant EW protein. Risk for clinical reaction to RE or SHE.
Gal d 3 (Ovotransferrin or Conalbumin)	76–77	Transferrin	Iron-binding capacity with antimicrobial activity	Low	Heat-labile. Risk for clinical reaction to RE or SHE.
Gal d 4 (lysozyme)	14.3	Glycoside hydrolase family 22	Antibacterial activity	Moderate	Risk for clinical reaction to RE or SHE.
Gal d 5 (α-livetin)	65–70	Serum albumin	Ions-binding, fatty acids, hormones in physiological conditions	n.a.	-

(EAACI: European Academy of Allergy and Clinical Immunology; HE: hen’s egg; n.a.: not applicable; RE: raw egg; SHE: slightly heated egg).

**Table 2:** A summary of the main studies conducted in the field of OIT with BE published from 2014 to 2024.

Reference	Population	Study Design	Outcome
Bravin et al., 2016 [69]	15 subjects (9 [60%] male), median age at the time of enrollment, 11 years and 2 months (range, 6–17 years).	Clinical trial	To evaluate the efficacy and safety of a recipe for an HE-containing biscuit to be used in home-based BE OIT for children with persistent HEA.
Gotesdyner et al., 2019 [70]	119 subjects divided into two groups: - Intervention group: 39 subjects (21 [53.8%] male), median age at the time of enrollment, 16 months (range, 0–2 years); - Control group: 80 subjects (51 [63.7%] male), after 2 years of age at the time of enrollment.	Case-control study	To evaluate the efficacy and safety of an SGEP with EHBE in promoting tolerance in children <2 years old with HEA.
Bird et al., 2019 [71]	13 subjects (9 [70%] male), median age at the time of enrollment, 4.3 years (range, 1.5–11.7 years).	Clinical trial	To determine if BE-allergic children would become desensitized to 6 g of LCE (e.g., scrambled) after undergoing BE OIT for approximately 2 years.
Pérez-Quintero et al., 2020 [72]	70 subjects divided into three groups: - BE-tolerant with BE ingestion: 33 subjects (18 [54.5%] male), median age at the time of first reaction, 12 months (range, 5–24 months); - BE-tolerant with BE exclusion diet: 16 subjects (10 [62.5%] male), median age at the time of first reaction, 12 months (range, 4–24 months); - BE non-tolerant: 21 subjects (10 [62.5%] male), median age at the time of first reaction, 12 months (range, 7–24 months).	Retrospective study	To determine if daily ingestion of BE would accelerate tolerance to RE in BE-tolerant patients compared to patients who tolerated BE at diagnosis but eliminated it from their diet and to patients who did not tolerate it.
Machinena et al., 2020 [73]	60 subjects (33 [55%] male), median age at the time of enrollment, 38.5 months (range, 1–6 years).	Prospective study	To evaluate tolerance to BE in HE-allergic children younger than 6 years.
Gruzelle et al., 2021 [74]	71 subjects (48 [67.6%] male), median age at the time of first OFC, 6 years (range, 2–17 years).	Retrospective study	To evaluate the efficacy and safety of BE OIT in children with HEA after a low-dose BE OFC.
Thomas et al., 2021 [75]	47 subjects, (32 [68%] male), mean age at the time of commencing HE ladder, 40 months (range, 0–18 years).	Retrospective study	Primary outcome: to evaluate the use of a structured HE ladder with regards to its safety as well as user satisfaction and barriers that arose in negotiating it. Secondary outcome: to determine potential risk factors which increased the likelihood of clinical reaction to foods containing HE and rates of eventual tolerance to LCE and RE achieved in the home environment.
De Vlieger et al., 2022 [76]	78 subjects, divided into two arms: - Short arm: 39 subjects (27 [%] male), median age at the time of enrollment, 3 years (range, 2–5 years); - Long arm: 39 subjects (23 [%] male), median age at the time of enrollment, 2 years (range, 1–3 years).	Prospective randomized intervention trial	To determine the optimal duration for gradually introducing HE tolerance at home in BE-tolerant children, with the goal of achieving RE tolerance.
Kotwal et al., 2023 [77]	243 subjects (157 [64.6%] male), median age at the time of OFC, 5 years (range, 6 months to 21 years).	Retrospective study	To characterize predictors of BE tolerance and progression to less-cooked forms of HE, as well as adverse reactions to home introduction of BE in a real-world setting.

(BE: baked egg; EHBE: extensively heated and baked egg; HE: hen’s egg; HEA: hen’s egg allergy; LCE: lightly cooked egg; OFC: oral food challenge; OIT: oral immunotherapy; RE: raw egg; SGEP: structured graduated exposure protocol).

## Data Availability

No new data were created or analyzed in this study.
